# NSC30049 inhibits Chk1 pathway in 5-FU-resistant CRC bulk and stem cell populations

**DOI:** 10.18632/oncotarget.19778

**Published:** 2017-08-01

**Authors:** Satya Narayan, Aruna S. Jaiswal, Ritika Sharma, Akbar Nawab, Lizette Vila Duckworth, Brian K. Law, Maria Zajac-Kaye, Thomas J. George, Jay Sharma, Arun K. Sharma, Robert A. Hromas

**Affiliations:** ^1^ Department of Anatomy and Cell Biology, University of Florida, Gainesville, FL 32610, USA; ^2^ Department of Medicine, University of Florida, Gainesville, FL 32610, USA; ^3^ Department of Pathology, Immunology and Laboratory Medicine, University of Florida, Gainesville, FL 32610, USA; ^4^ Department of Pharmacology and Experimental Therapeutics, University of Florida, Gainesville, FL 32610, USA; ^5^ Celprogen, Inc., Torrance, CA 90503, USA; ^6^ Department of Pharmacology, Penn State Cancer Institute, Penn State College of Medicine, Hershey, PA 17033, USA

**Keywords:** colorectal cancer, novel compound, FOLFOX-resistance, CRC stem cells, replication stress

## Abstract

The 5-fluorouracil (5-FU) treatment induces DNA damage and stalling of DNA replication forks. These stalled replication forks then collapse to form one sided double-strand breaks, leading to apoptosis. However, colorectal cancer (CRC) stem cells rapidly repair the stalled/collapsed replication forks and overcome treatment effects. Recent evidence suggests a critical role of checkpoint kinase 1 (Chk1) in preventing the replicative stress. Therefore, Chk1 kinase has been a target for developing mono or combination therapeutic agents. In the present study, we have identified a novel orphan molecule NSC30049 (NSC49L) that is effective alone, and in combination potentiates 5-FU-mediated growth inhibition of CRC heterogeneous bulk and FOLFOX-resistant cell lines in culture with minimal effect on normal colonic epithelial cells. It also inhibits the sphere forming activity of CRC stem cells, and decreases the expression levels of mRNAs of CRC stem cell marker genes. Results showed that NSC49L induces 5-FU-mediated S-phase cell cycle arrest due to increased load of DNA damage and increased γ-H2AX staining as a mechanism of cytotoxicity. The pharmacokinetic analysis showed a higher bioavailability of this compound, however, with a short plasma half-life. The drug is highly tolerated by animals with no pathological aberrations. Furthermore, NSC49L showed very potent activity in a HDTX model of CRC stem cell tumors either alone or in combination with 5-FU. Thus, NSC49L as a single agent or combined with 5-FU can be developed as a therapeutic agent by targeting the Chk1 pathway in 5-FU-resistant CRC heterogeneous bulk and CRC stem cell populations.

## INTRODUCTION

The development of CRC is the result of not just one, but the accumulation of many genetic epigenetic alterations [1]. Following surgery for advanced colorectal cancer (CRC), only 20–30% patients respond to standard chemotherapy [2]. Despite attempts to improve patient outcomes by incorporating new active systemic agents into clinical practice, there has been little improvement in the metastatic CRC patient cure rate. In spite of the best practice of 5-fluorouracil (5-FU), with or without additional therapy to eradicate micrometastatic disease after “curative” surgery for early stage CRC or in those with oligometastatic disease, most cancers relapse within the first few years following treatment completion [3, 4]. This indicates a relatively rapid repopulation of neoplastic progeny, i.e., CRC stem cells [5, 6]. 5-FU is the most integral systemic component of curative and palliative therapy for CRC, but overall response rate in advanced CRC is only 10–15%. In modern clinical practice, the combination of 5-FU with leucovorin and oxaliplatin (FOLFOX) has demonstrated improved response rates [3]. However, these tumors quickly develop resistance to FOLFOX [7], and at the same time, this treatment regime leads to significant toxicity, cost, and patient inconvenience [3].

The traditional view of cancer development suggests that all neoplastic cells within a tumor contain tumorigenic growth capacity [8–10]. However, only a small subset of CRC bulk cells is able to initiate tumor growth. This subset of cells is identified as CRC-initiating cells or CRC stem cells [11]. Thus, to optimally treat CRC, a therapy is required that can target both CRC heterogeneous bulk and stem cell populations and also overcome 5-FU resistance. To achieve this goal, a mechanism-based systemic therapy with a well-characterized specific target is needed to eliminate the neoplastic progeny [12, 13] and improve clinical outcomes.

To address these concerns, we began screening orphan molecules with desired biological activity against CRC. We found one such compound named NSC30049 (1-(4-Chloro-2-butenyl)-1λ∼5∼,3,5,7-tetraazatricyclo [3.3.1.1∼3,7∼] decane). NSC30049 qualifies as a drug-like compound with Lipinski score 4, molecular weight 229.73, H-bond donors 0, and H-bond acceptors 3 [14, 15]. This compound has not been tested before for its biological activity against cancer cells, except one non-cancer related study that was conducted against human immunodeficiency virus (HIV) strain 1(RF) by the Developmental Therapeutics Program (DTP)-National Cancer Institute (NCI). The results confirmed that NSC30049 was inactive against HIV (http://dtp.cancer.gov/dtpstandard/AIDSData/index.jsp). We sought to investigate the anti-neoplastic and mechanistic properties of this compound as a potential adjunct to standard treatments.

In the present study, we found that NSC30049 is a highly active molecule with potent toxicity to CRC cell lines, but is much less toxic to a normal colonic epithelial cell line. We show that NSC30049 has characteristics of a Chk1 inhibitor. It has potent cytotoxic effect on FOLFOX-resistant CRC cells, as well as CRC stem cells *in vitro*. NSC30049 is readily soluble in water with no toxicity to animals when used below the maximum tolerated dose (MTD). The pharmacokinetic analysis of NSC30049 suggests its high bioavailability, but with shorter plasma half-life. In patient-derived tumor xenograft (PDTX) studies, NSC30049 alone and in combination with 5-FU dramatically reduced the growth of CRC stem cell tumors in mice. Thus, these studies provide evidence for developing the future drug for the treatment of resistant CRC tumors either alone or in combination with 5-FU to ultimately reduce both morbidity and mortality from CRC. Since NSC30049 has not been developed by pharmaceutical industries and has usefulness for treating FOLFOX-resistant CRC tumors, as well as targeting CRC stem cells, it may fall in the category of an orphan drug molecule beneficial to public health.

## RESULTS AND DISCUSSION

### *Trans*-NSC30049 has higher growth inhibitory effect on HCT116 cells than *cis-*NSC30049

**Figure 1:**
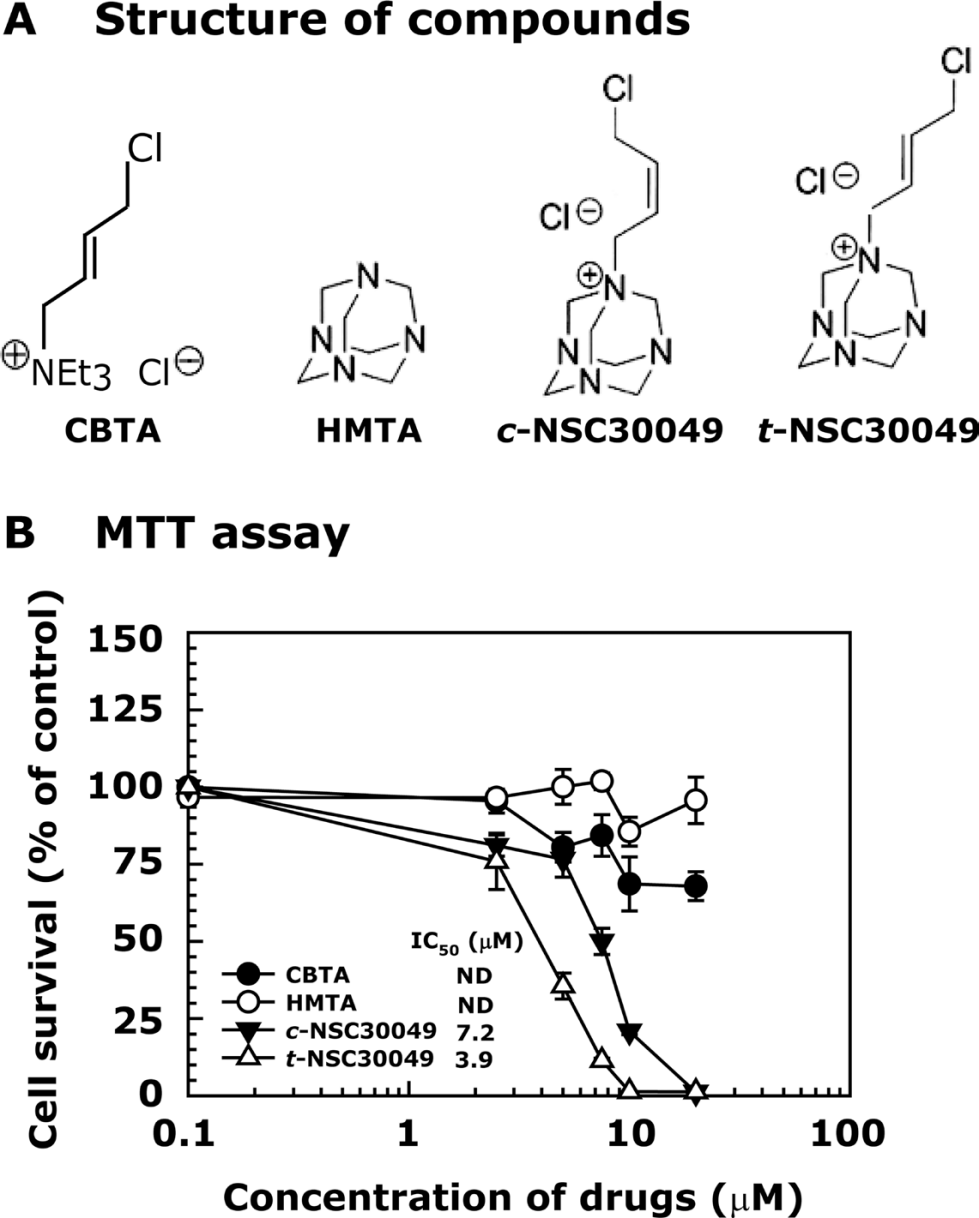
Changing from *cis*- to *trans*-position of the double bond in NSC30049 increases its biological activity Panel (**A**) represents the structure of compounds. Panel (**B**) shows the effect of compounds on the survival of CRC cell lines. Cells were grown in 96-well plates, treated with different concentrations of compounds for 48 h. The survival of the cells was determined by MTT assay. Data are representative of mean ± SE of three different experiments.

We synthesized *cis*-NSC30049 (*c-*NSC30049) and *trans*-NSC30049 (*t-*NSC30049) in our laboratory [16]. We tested their biological activity in a cell survival experiment (MTT-assay) against HCT-116 cells. First, to understand whether the halogen group (Cl^−^), a suspected DNA-reactive group, and the ring structure of the NSC30049 indecently carry the growth inhibitory activity, we used (4-Chloro-but-2-enyl)triethylammonium chloride (CBTA) and hexamethylenetetramine (HMTA), for the synthesis of NSC30049 (Figure 1A) [16]. Results show that CBTA and HMTA have very little cytotoxic effect, suggesting that the halogen group of CBTA and the ring structure of the HMTA alone may not be sufficient for the cytotoxic effect of NSC30049. Second, we determined the stereoisomeric effect of NSC30049 on the survival of HCT-116 cells. We found that *t-*NSC30049 was more active than *c-*NSC30049 (IC_50_ 3.9 and 7.2 μM, respectively) (Figure 1B). Since *t*-NSC30049 – from here onward called as NSC49L (where L denotes for the lead compound) – has better potency than *c-*NSC4009, we used NSC49L in our further studies.

### NSC49L inhibits the growth of CRC cell lines from different genetic backgrounds and enhances the cytotoxic effect of 5-FU

The development of CRC takes place due to the accumulation of mutations in oncogenes (K-*ras*, PI3K, B*-raf and β-catenin*), tumor suppressor (*APC, p53* *and Smad4*) genes, and DNA repair genes [17]. In these experiments, we tested whether NSC49L treatment alone or in combination enhances the 5-FU effect of inhibiting the growth of CRC heterogeneous bulk cell lines with different genetic backgrounds, as indicated in Table 1. In these cell lines, we determined the IC_50_ of NSC49L and 5-FU alone or in combination. Our results show that NSC49L alone inhibits the growth of all the CRC heterogeneous bulk cell lines tested irrespective of their different genetic backgrounds (Table 1). Moreover, the effect of NSC49L on a normal human colonic epithelial cell line, HCoEpiC, was extremely low as compared to the CRC heterogeneous bulk cell lines (Table 1). Furthermore, when we combined NSC49L with 5-FU at low concentrations, the IC_50_ of 5-FU was significantly reduced (Table 1). These results indicate that NSC49L can reduce the effective dose of 5-FU and inhibit the growth of CRC heterogeneous bulk cell lines with MMR-deficiency carrying K-*ras*/B-*raf*/β-catenin mutations. 5-FU inhibits thymidylate synthase activity, reducing the size of available deoxyribonucleotide triphosphate (dNTP) pools that are needed for DNA synthesis, thus enhancing replicative stress as a mechanism of cellular toxicity of rapidly perforating cancer cells [18].

**Table 1:** IC_50_ of NSC49L and 5-FU treated either alone or in combination to normal colonic epithelial and CRC cell lines

Treatment	HCoEpiC Normal colonic Epithelial cell line	HCT-116 p53*^wt^* MMR^−^ K-ras*^mut^* β-catenin*^mut^*	LoVo p53*^wt^* MMR^−^ K-ras*^mut^* β-catenin*^mut^*	SW480 p53*^mut^* MMR^+^ K-ras*^mut^* β-catenin*^wt^*	LS174T p53*^wt^* MMR^−^ K-ras*^mut^* β-catenin*^wt^*	RKO p53*^wt^* MMR^−^ B-raf*^mut^* β-catenin*^wt^*
	IC_50_ (μM)					
NSC49L	31.9 ± 0.9	2.8 ± 0.5	4.2 ± 0.1	4.4 ± 0.2	4.6 ± 0.8	4.9 ± 0.4
5-FU	513 ± 22	11.5 ± 1.5	22.0 ± 0.9	113 ± 6	51 ± 3.8	68 ± 10.4
5-FU+1.5 μM NSC49L	446 ± 56	9.2 ± 0.9 *	10.4 ± 2.0 *^,·^	12.3 ± 2.8 *^,·^	4.9 ± 0.1 ^·^	24.7 ± 3.3 *^,·^

For these experiments, 1,500 live cells were seeded in 96-well plates and treated with different concentrations of drugs either alone or in combination for 48 h. The survival of the cells was determined by MTT assay. Data are mean ± SE of four estimations. * and ^·^ = Significantly different as compared to NSC49L and 5-FU, respectively, *p* < 0.05. *wt* = wild-type, *mut* = mutant, MMR^−^ = mismatch repair deficient, and MMR^+^ = mismatch repair proficient.

### NSC49L inhibits the growth of 5-FU-resistant HCT-116 and HT29 cell lines and CRC stem cell sphere formation ability *in vitro*

**Figure 2:**
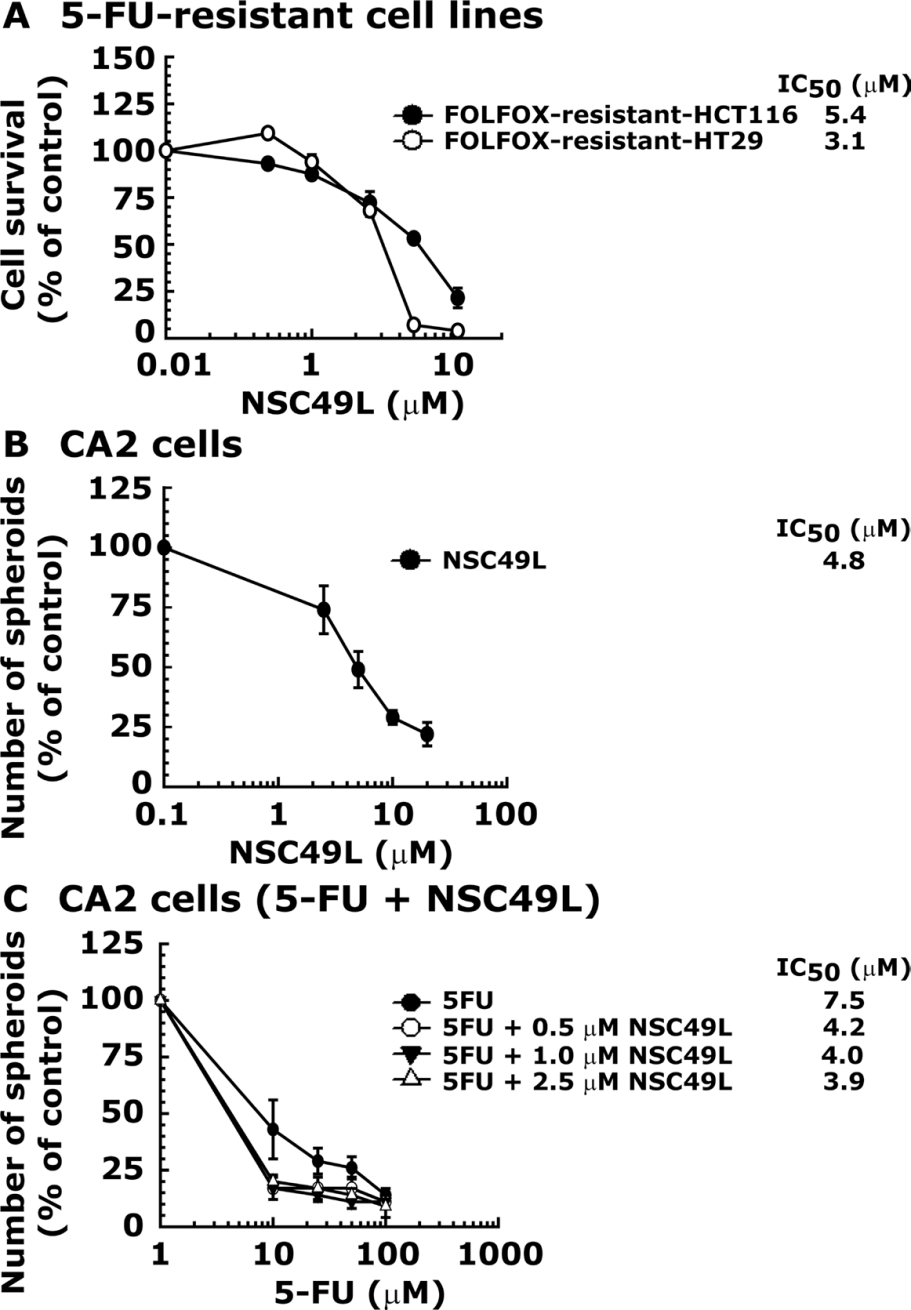
NSC49L sensitizes FOLFOX-resistant-HCT116 and FOLFOX-resistant-HT29 cell lines and decreases the sphere formation capacity of CA2 stem cells Panel (**A**) is the MTT assay. FOLFOX-resistant-HCT116 and FOLFOX-resistant-HT29 Cell lines were treated with different concentrations of NSC49L for 48 h. The survival of the cells was determined by MTT assay. The IC_50_ data are the mean ± SE of four estimations. Panel (**B**) represents the sphere formation assay. CA2 cells were treated with different concentrations of NSC49L and the sphere formation efficiency was determined on the seventh day. Data are mean ± SE of four different estimations. Panel (**C**) shows the effect of NSC49L on the sphere formation capacity of CA2 cells when treated in combination with 5-FU. Data are mean ± SE of four different estimations.

The failure of FOLFOX treatment against relapsed CRC tumors is due to the development of resistance against these drugs [7]. Therefore, we determined whether NSC49L can sensitize FOLFOX resistant CRC tumors. In these studies, we used FOLFOX-resistant CRC heterogeneous bulk cell lines HCT-116 and HT29 [19]. The results showed that FOLFOX-resistant-HCT116 and FOLFOX-resistant-HT29 cell lines were sensitized with NSC49L treatment with an IC_50_ of 5.4 μM and 3.1 μM, respectively (Figure 2A). These results suggest that NSC49L can be a future choice for treatment of FOLFOX-resistant CRC tumors.

Further, since the clinical relapse of CRC is often associated with progeny repopulation from resistant CRC stem cells [5, 6, 11], we then examined whether NSC49L and 5-FU can sensitize these cells in sphere formation assays. For these studies, we used a well-characterized CRC stem cell line, CA2 [20, 21]. We performed sphere formation assays involving a three-dimensional (3D) culture system in serum-free conditions to determine whether NSC49L alone or in combination with 5-FU can inhibit the sphere formation ability of CA2 cells. Results showed that NSC49L inhibited the growth of spheres better than 5-FU (Figure 2B and 2C). Furthermore, the combination of NSC49L with 5-FU reduced the IC_50_ of this drug in a dose-dependent manner (Figure 2C). These results suggest that NSC49L can inhibit the growth of CA2 cells when treated alone and can also increase the efficacy of 5-FU when used in combination.

### NSC49L inhibits the expression of self-renewal genes in CRC stem cells more potently than 5-FU

**Figure 3:**
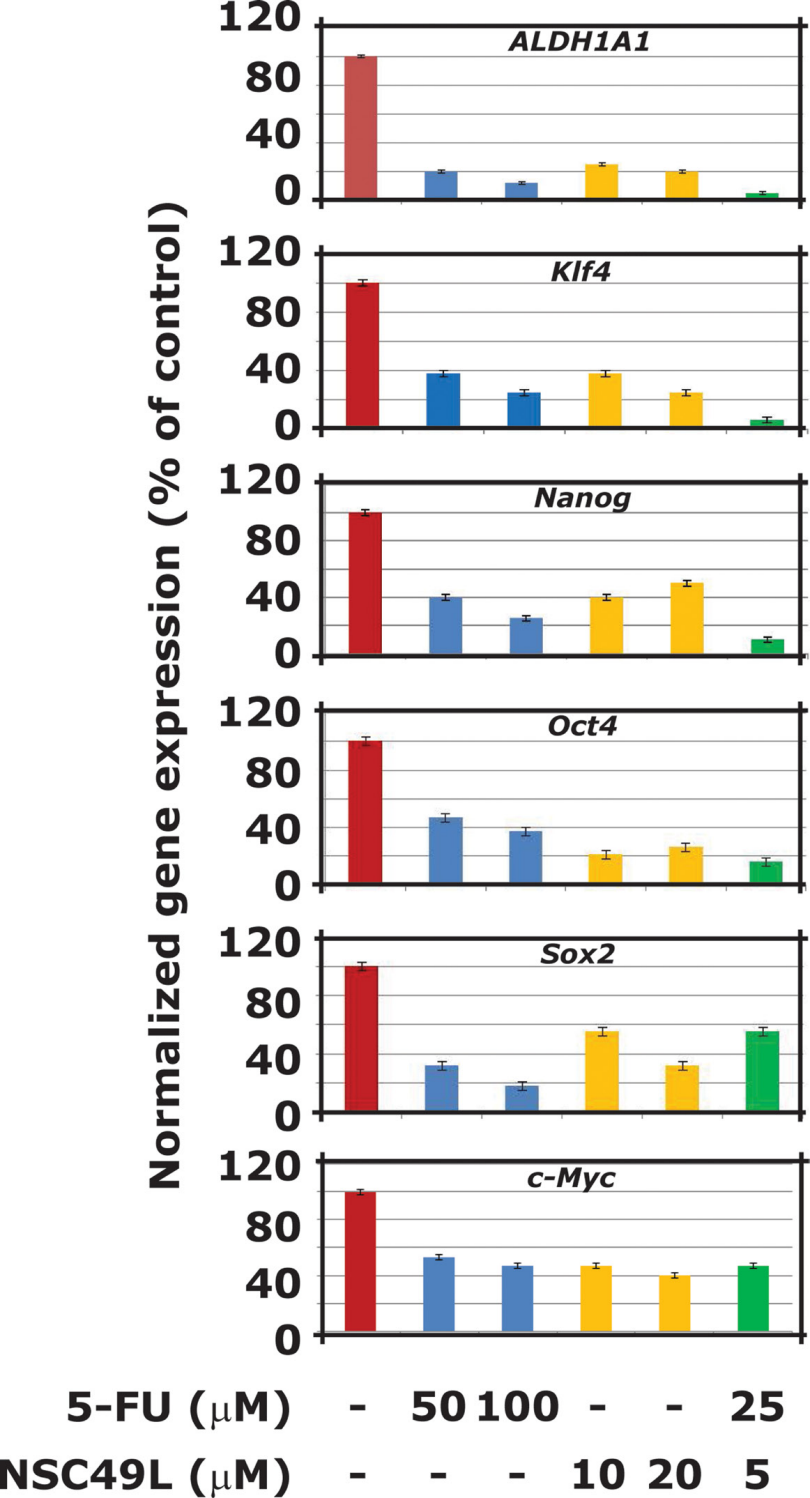
Effect of NSC49L and 5-FU either alone or in combination on the mRNA levels of key marker and self-renewal genes For these experiments, CA2 cells were treated with different concentrations of NSC49L and 5-FU either alone or in combination for 72 h. Total RNA was isolated and the expression levels of different genes was determined by qRT-PCR. The expression was normalized with *GAPDH* mRNA levels. Data are mean ± SE of three different estimations.

CRC stem cells express marker and self-renewal genes, such as *ALDH1*, *KLF4*, *Nanog*, *Oct4*, *Sox2*, and *c-Myc* [20–22]. The overexpression of these genes is often considered as chemoresistance markers for cancer stem cells [23, 24]. We determined whether the NSC49L-induced decrease in sphere forming ability of CA2 cells is linked with decreased expression of these marker and self-renewal genes. Results showed that the mRNA levels of all these genes were decreased by both NSC49L as well as 5-FU treatments. However, when NSC49L treatment was combined with 5-FU the mRNA levels of *ALDH1*, *KLF4* and *Nanog* were further decreased in these cells (Figure 3). These results suggest that the stemness characteristics of CA2 cells were decreased by NSC49L treatment with and the combination of 5-FU results were even more pronounced. Since overexpression of *ALDH1A1, KLF4* and *Nanog* is an indicator of CRC stem cells, other malignancies, and targets for combined chemotherapy [20, 25–31], the decreased expression of these genes by NSC49L may prove a useful therapeutic agent for the intervention of CRC progression by targeting to CRC stem cells.

### NSC49L enhances hydroxyurea (HU)-induced S-phase arrest of HT29 cells

In cancer cells, replicative stress is a mechanism for the perturbation of error-free DNA replication, decreased DNA synthesis, increased genomic instability, S-G_2_/M-phase arrest, and tumorigenesis [32, 33]. However, by enhancing replicative stress through further perturbing S-G_2_/M checkpoints in cancer cells, a mitotic catastrophe can be induced, with accumulated single-stranded DNA (ssDNA) and double-stranded DNA (dsDNA) breaks, that exceeds the repair capacity of the cell; and leads to cell death [33–35]. Recently, this idea has been employed in clinical studies for therapeutic developments [36]. Since 5-FU is known to induce replication stress as one of the mechanisms for its chemotherapeutic activity [37, 38], and several inducers of the replication stress pathways have been studied [18, 38, 39], we examined whether NSC49L may also function through induced replication stress and S-G_2_/M phase arrest.

**Figure 4:**
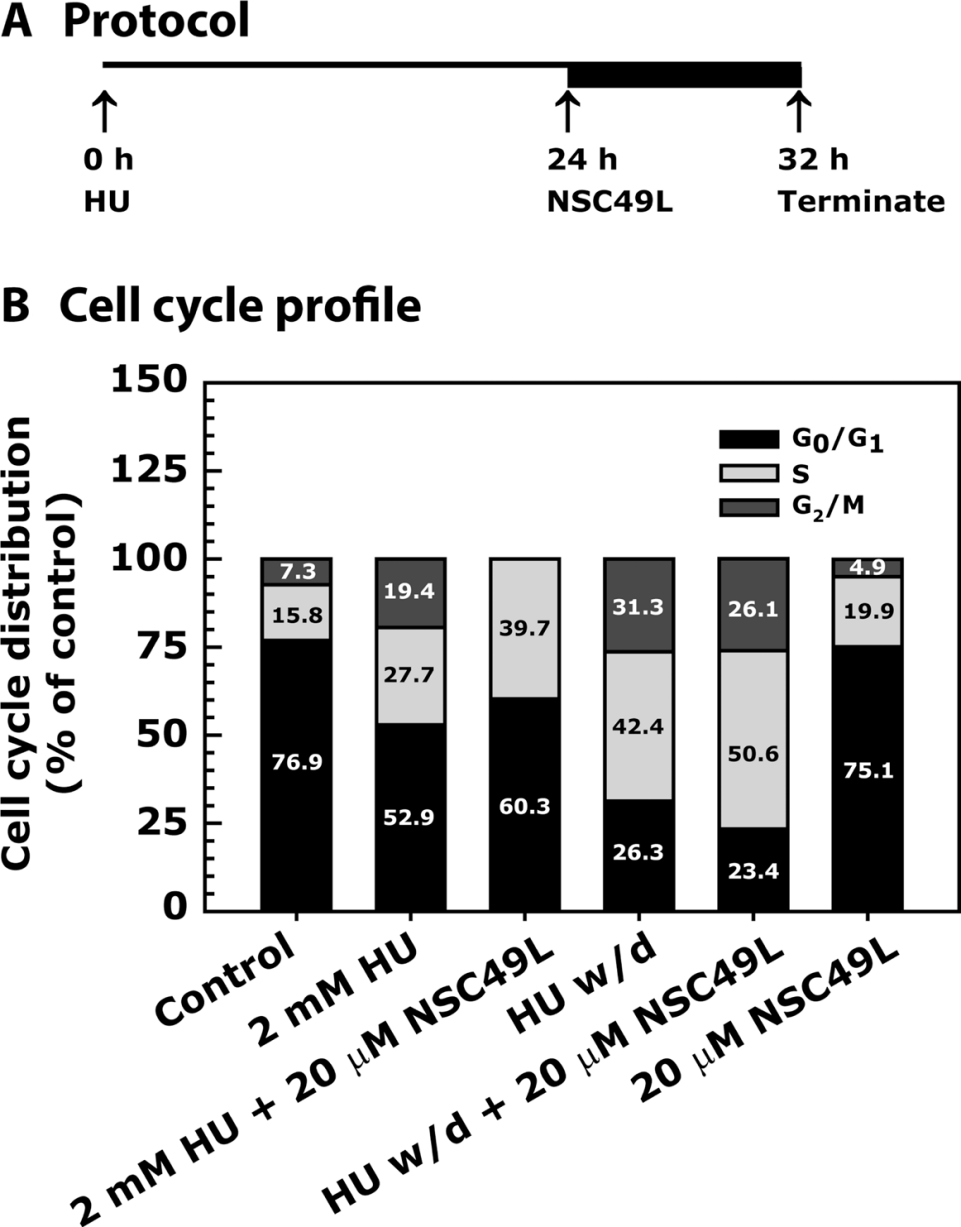
Cell cycle profile of HT29 cell line-treated with NSC49L and HU either alone or in combination Panel (**A**) describes the assay protocol. Panel (**B**) shows the data as a stacked bar graph. Cells were grown in 0.5% FBS for 20 h and then treated with 2 mM HU for 24 h. Then, either in the presence or absence of HU, cells were treated with 20 μM NSC49L for an additional 8 h. After treatment, cells were processed for cell cycle analysis by fluorescence-activated cell sorting (FACS). The ranges for G_0_/G_1_, S and G_2_/M-phase arrested cells were established on the basis of the corresponding DNA content of the histograms. At least 10,000 cells per sample were considered in the gated regions for calculations. Data are mean percent distribution of DNA content of two different experiments.

Since hydroxyurea (HU), an inhibitor of ribonucleotide reductase (RNR) that disrupts the metabolism of dNTPs [40, 41], is a pure inducer of replication stress-dependent S-phase arrest [42, 43] than 5-FU that has activities beyond S-phase. We used this agent in mechanistic studies to determine whether NSC49L can further induce HU-mediated S-phase arrest. Since most of the human cancer cells harbor defective G1 checkpoint due to mutations in *p53* gene [44], they become more dependent upon S-phase and G2-phase kinases, mainly to Chk1, to induce cell cycle arrest in response to DNA damage. Therefore, we used p53 mutant HT29 cell line to examine the effect of NSC49L on replicative stress and S-phase arrest without the interference of the p53 signaling. HT29 cells were treated with HU either alone or in combination with NSC49L as indicated in Figure 4A. A 24 h treatment with 2 mM of HU caused 27.7% S-phase and 19.4% G_2_/M-phase arrest of HT29 cells. When cells were further treated with 20 μM of NSC49L for additional 8 h, the S-phase arrest increased to 39.7% and there was complete blockage to the entry of G_2_/M-phase (Figure 4B). After 24 h treatment, when HU was withdrawn and the incubation of cells was continued for additional 8 h in drug-free medium, the S-phase arrest increased to 42.4% as compared to only 24 h HU treatment, suggesting that HU-induced replicative stress is persistent and exists for a longer period (Figure 4B). Further, when HT29 cells were treated with NSC49L for 8 h after the withdrawal of the HU, the S-phase arrest increased to 50.6% with 26.1% of the cells in G_2_/M-phase, suggesting a combined effect of NSC49L and HU on the S-phase arrest (Figure 4B). Treatment with 20 μM of NSC49L alone showed a similar cell cycle profile as the untreated control cells (Figure 4B), suggesting that NSC49L alone may have no effect on cell cycle progression. These results indicate that NSC49L induces HU-mediated S-phase arrest of HT29 cells that may be due to increased replicative stress, leading to DNA damage accumulation and cell death.

### NSC49L treatment inhibits HU-induced Chk1 phosphorylation in HT29 cells

**Figure 5:**
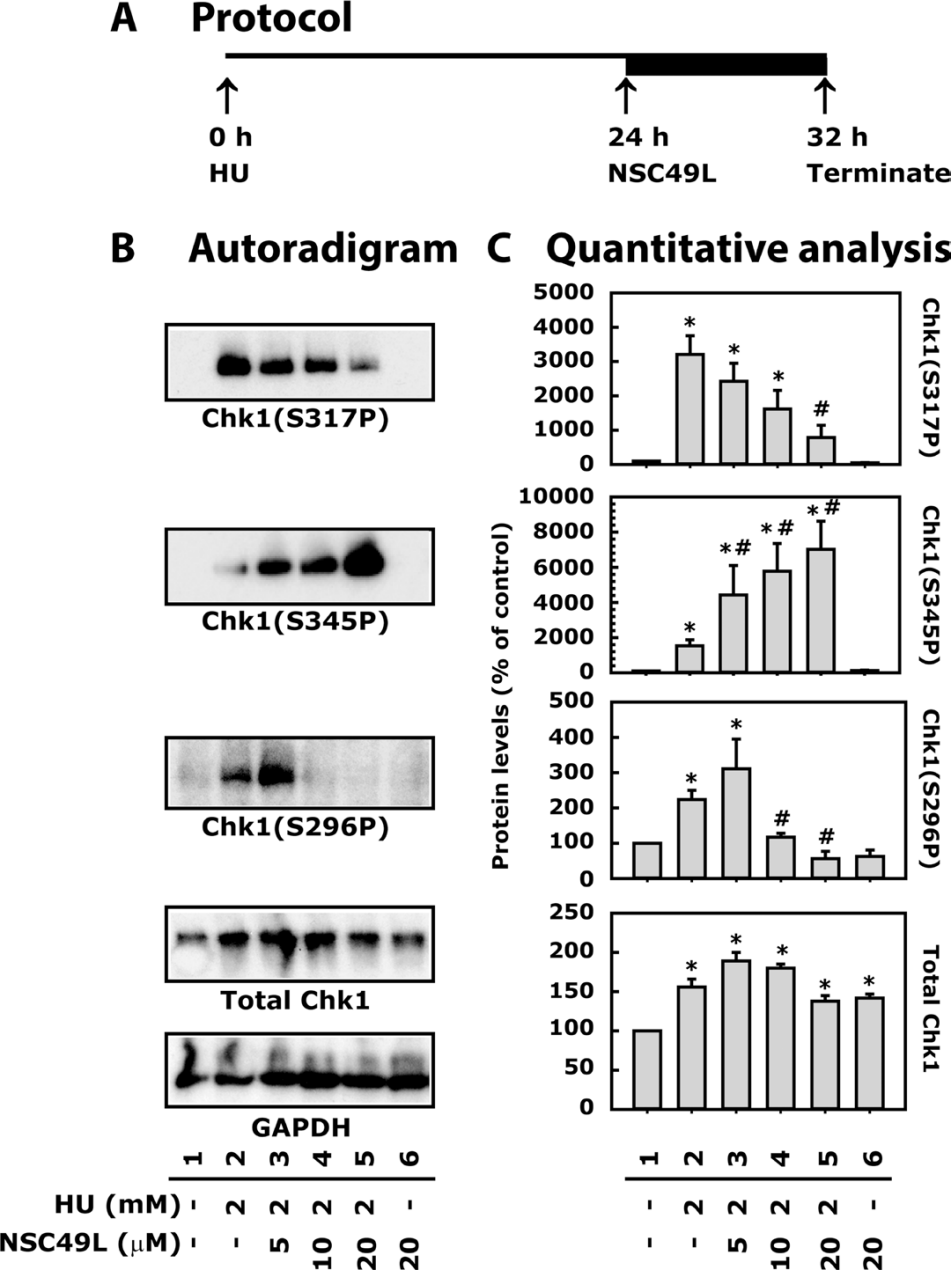
Treatment of NSC49L and HU differentially regulate total and phosphorylated levels of Chk1 in HT29 cells Panel (**A**) depicts the protocol of the assay. Cells were treated with 2 mM of HU for 24 h followed by different concentrations of NSC49L for 8 h. Panel (**B**) shows representative autoradiograms of Chk1 Western blots analyzed with whole cell extract. Normalization of protein loading was assessed by GAPDH. Panel (**C**) is the quantitative analysis of autoradiograms. Data are mean ± SE of three different experiments.

The reduced dNTPs metabolism after HU treatment induces replication stress that blocks the progression of replication forks [45], leading to the accumulation of ssDNA [33–35]. The presence of ssDNA activates the ATR/Chk1 signaling pathway [46, 47]. The ATR-mediated activation of Chk1 is dependent upon TopBP1 and Claspin. TopBP1 binds to replication protein A (RPA) at the ssDNA via 9-1-1 complex (PCNA-like RAD9/RAD1/HUS1 checkpoint clamp) [48], and stimulates ATR activity [49, 50]. Then Claspin interacts with this complex and goes through ATR-dependent phosphorylation [51]. The phosphorylated Claspin recruits Chk1 to this complex that is phosphorylated by ATR [52, 53]. Chk1 gets activated after phosphorylation by ATR at S317 and S345 [54, 55], and by auto-phosphorylation at S296 [56]. In the present experiments, treatment with 2 mM HU for 24 h increased phosphorylation of Chk1 at S317, S345 and S296 (Figure 5B, lane 2). However, after 24 h HU treatment when NSC49L treatment was combined for additional 8 h, as shown in Figure 5A, the Chk1(S317P) level was decreased in a dose-dependent manner (Figure 5B and 5C, compare lane 2 with 3–5, respectively). The Chk1(S296P) levels increased at low concentrations of NSC49L (5 μM), but decreased at 10 and 20 μM concentrations (Figure 5B and 5C, compare lane 2 with 3, 4 and 5, respectively). However, the level of Chk1(S345P) was increased above the HU treatment level in a dose-dependent manner when NSC49L was combined with HU treatment (Figure 5B and 5C, compare lane 2 with 3–5, respectively). Treatment with NSC49L alone did not show any significant effect on Chk1 phosphorylation, except there was some increase in the level of total Chk1 (Figure 5B and 5C, compare lane 1 with 6).

Generally, after DNA damage, inhibition of Chk1(S317P) and Chk1(S296P) abrogates S-phase progression [57, 58]. However, in our experiments this was not the case. Instead, we noted increased S-phase arrest in HU and NSC49L combination treatments. Since HU is a RNR inhibitor, there remains no more dNTPs that can be incorporated into DNA to force cells into G_2_-phase. Thus, the toxicity of HU plus NSC49L could be due to an increased permanent DNA damage burden in S-phase that goes beyond the repair capacity of the cell. In previous studies, an increased DNA damage burden effect was observed after treatment of breast cancer cell line MDA-MB-231 with the antimetabolite gemcitabine and the Chk1 inhibitor MK-8776 [59].

Furthermore, Chk1(S345P) levels were decidedly increased in a dose-dependent manner after NSC49L was combined with HU treatment (Figure 5B and 5C, compare lane 2 with 3–5, respectively), suggesting an increased level of DNA damage. However, the decreased Chk1(S317P) and Chk1(S296P) levels indicate the inhibition of Chk1 activity after HU and NSC49L treatment. These effects are similar with the treatment of Chk1 inhibitor SCH900776 in combination with HU [60]. To further support our findings that NSC49L may act as a potent Chk1 inhibitor, in previous studies, the treatment of CRC cell lines with different DNA damaging agents, including gemcitabine, showed decreased phosphorylation of Chk1 at S317 and S296, but increased phosphorylation at S345 after combination treatment with Chk1 inhibitor V158511 [61]. The increased Chk1(S345P) level has also been considered as a pharmacodynamic biomarker of gemcitabine and Chk1 inhibitor, ZD7772. These treatments are linked with an increased DNA damage response to chemotherapy [62]. Similarly, treatment with gemcitabine and the Chk1 inhibitor, MK8776, has been shown to induce S345P levels and decrease S296P levels of Chk1, suggesting greater DNA damage in tumor cells versus normal cells [63].

In recent studies, a regulatory circuit of ATR/Chk1/phosphatase 2A (PP2A) has been suggested to regulate Chk1 phosphorylation in cells. ATR continually phosphorylates Chk1 on S317 and S345 residues. The phosphorylated Chk1(S317P) and Chk1(S345P) either directly or indirectly stimulates PP2A to dephosphorylate Chk1 at S317 and S345 sites [64]. Therefore, through this feedback mechanism when Chk1 is inhibited, ATR may become active and phosphorylate Chk1 at S345 after HU and NSC49L treatments. Another Chk1(S345P) regulator is Wip1 phosphatase. It has been shown that overexpression of Wip1 resulted in the elimination of Chk1 phosphorylation at S345 and S317 [65], and Wip1-deletion reversed the process [66]. Whether the PP2A or Wip1 pathways are involved in HU/NSC49L-mediated Chk1phosphorylation at S345 is not known.

### NSC49L treatment alone induces Chk1 phosphorylation at S345 and inhibits S317 and S296 phosphorylation when combined with 5-FU in HCT-116 and CRC stem cell line CA2

**Figure 6:**
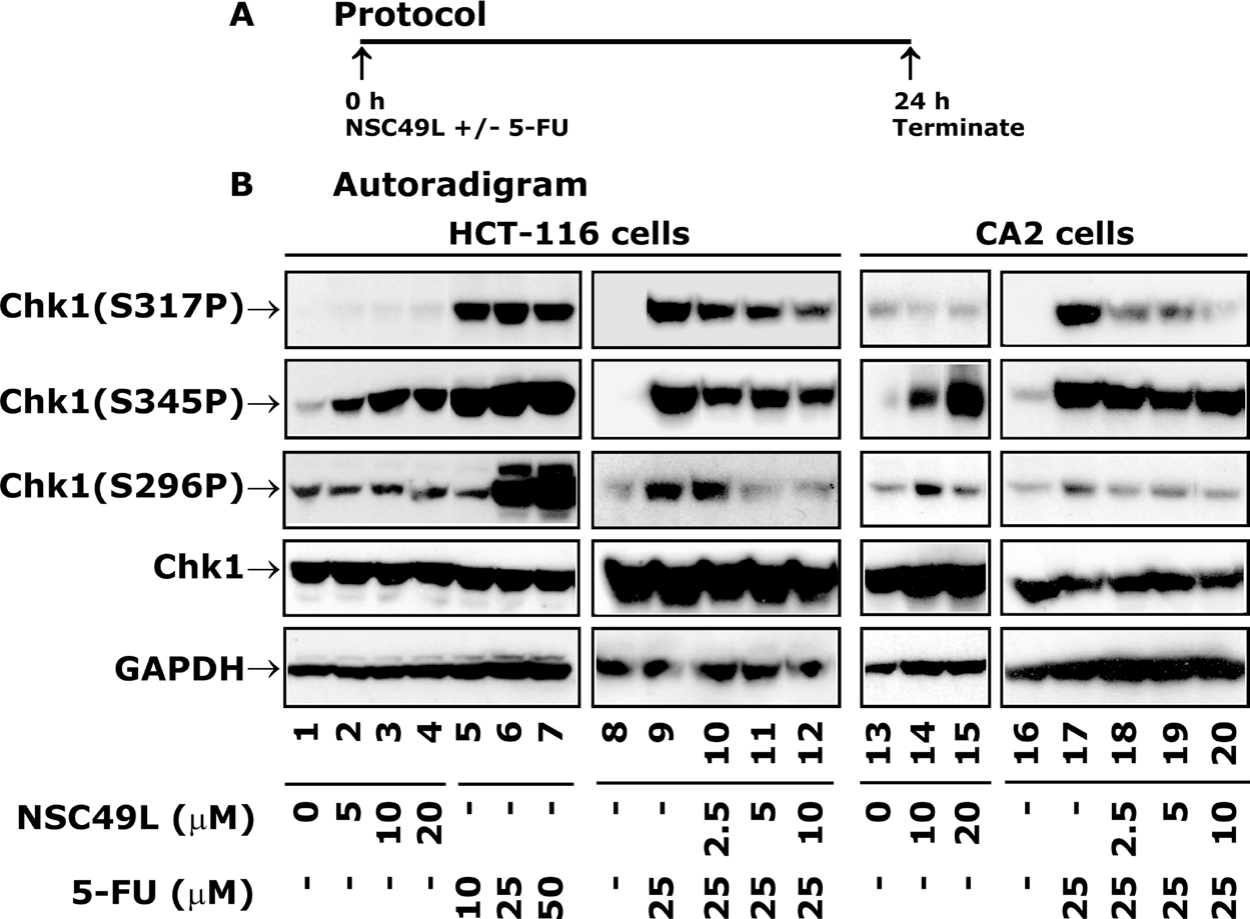
Expression levels of total and phosphorylated Chk1 in HCT-116 and CA2 cells after treatment with NSC49L and 5-FU Panel (**A**) depicts the protocol of the assay. Panel (**B**) shows autoradiograms of Western blots. Cells were treated with different concentrations of NSC49L and 5-FU either alone or in combination for 24 h. The whole cell extract was prepared and processed for Western blot analysis. Normalization of protein loading was assessed by GAPDH. Data are representative of three different experiments.

In these experiments, we determined the long-term treatment effect (24 h) of the NSC49L either alone or in combination with 5-FU to HCT-116 cells (Figure 6A). Interestingly, while an 8 h treatment with NSC49L alone to HT29 cells did not show any effect on Chk1 phosphorylation, there was a dose-dependent increase in the levels of Chk1(S345P) with no effect on the levels of Chk1(S317P) and Chk1(S296P) in HCT-116 cells (Figure 6B, compare lane 1 with 2–3). This differential effect of NSC49L treatment on Chk1 phosphorylation in HT29 and HCT116 cell lines could be due to the duration of the treatment or some other mechanism that is unknown at this moment. On the other hand, treatment with 5-FU alone increased phosphorylation at all three Chk1 sites in HCT-116 cells (Figure 6B, compare lane 1 with 5–7). Consistent with the idea that the increased phosphorylation of Chk1 at S345 can be a DNA damage marker [61–63], the increased level of Chk1(S345P) with NSC49L treatment for 24 h suggests that NSC49L alone affects the ATR/Chk1 pathway that can be developed as a successful single agent therapy. This is evident from its strong cytotoxic effect on different CRC cell lines (Table 1).

Similar to HCT116 cells, the Chk1(S317P) and Chk1(S296P) levels in CA2 cells showed very little change after NSC49L alone treatment for 24 h; however, an increase in a dose-dependent manner was observed with Chk1(S345P) level (Figure 6B, compare lane 13 with 14 and 15, respectively). We then determined the combined effect of NSC49L and 5-FU on CA2 cells after simultaneous treatment for 24 h. The combination treatment of HCT-116 and CRC stem cell line, CA2, with NSC459L and 5-FU resulted in decreased levels of Chk1 phosphorylation at S317 and S296, but not at S345 (Figure 6B, compare lane 9 with 10–12 and 17 with 18–20, respectively). The total Chk1 levels were not affected by these treatments (Figure 6B, compare lane 1 with 2–7, lane 8 with 9–12, lane 13 with 14 and 15, and lane 16 with 17–20, respectively). These results suggest that NSC49L may be inducing a DNA damage response of 5-FU through the inhibition of the Chk1 pathway.

### The toxicity of HU is much higher in HCT116(p53^+/+^) than HCT116(p53^−/−^) cells

**Figure 7:**
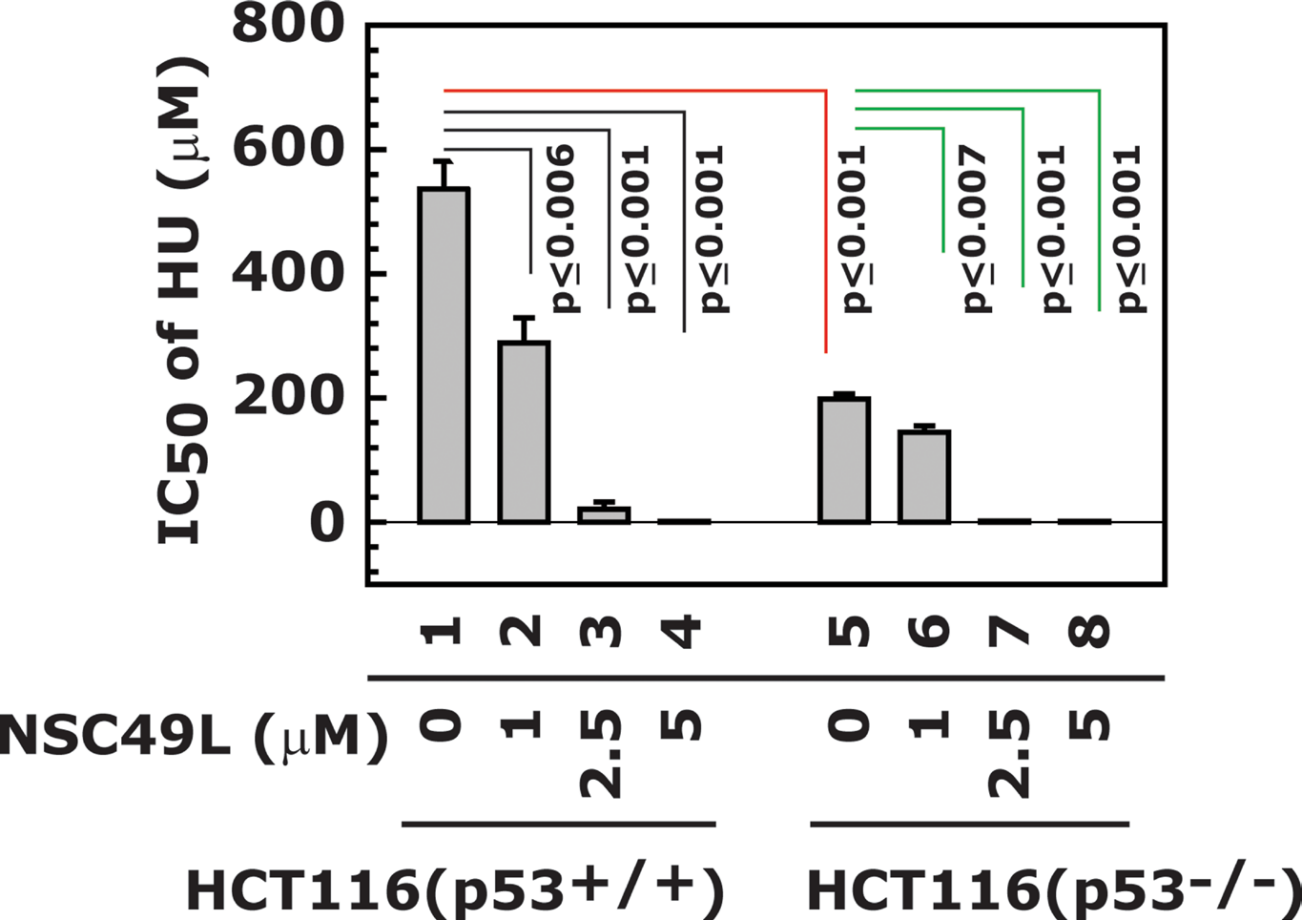
Synthetic lethal effect of NSC49L and HU treatment to HCT116(p53^−/−^) cells The isogenic HCT116(p53^+/+^) and HCT116(p53^−/−^) cell lines were treated with different concentrations of NSC49L and HU for 72 h in 96-well plates. The IC_50_ of HU was determined by MTT assay. Data are mean ± SE of three different experiments. The *p* value is given and compared with its comparative groups.

The importance of the p53 for ATR/Chk1 activity has been previously well-documented. A mouse model of Seckel syndrome – characterized by a severe deficiency in ATR – exhibits severe replicative stress that worsened with the loss of p53 and aging [67]. This finding was similar to those showing toxicity of Chk1 inhibitors to p53-deficient cancer cells [68]. In the present experiment, we determined whether NSC49L can enhance the cytotoxicity of HU in isogenic HCT116(p53^+/+^) and HCT116(p53^−/−^) cell lines. The results showed a decreased IC_50_ of HU in HCT116(^−/−^) compared with HCT116(p53^+/+^) cells (Figure 7, compare lane 1 with 5) that was further decreased by treatment with NSC49L (Figure 7, compare lane 1 with 2–4 and 5 with 6–8, respectively). These results suggest that HCT116(p53^−/−^) cells are more sensitive to HU and NSC49L treatments than HCT116(p53^+/+^) cells. This effect could be due to increased replicative stress-mediated DNA damage during S-phase in p53-deficient cells, as previously observed with Chk1 inhibitors [69, 70]. In recent studies, a combination treatment of Chk1 inhibitors with several therapeutic drugs has been in clinical trials for the treatment of patients with CRC, breast and other tumors, especially containing p53 mutations [71]. Furthermore, a similar toxicity for p53-deficient cells was also observed with an ATR inhibitor [72]. These studies support our findings that NSC49L has potential for development as a synthetic lethal compound in combination with 5-FU for inducing toxicity to p53-deficient CRCs.

### NSC49L and 5-FU treatments lead to double-strand DNA breaks in HCT-116 and CA2 cells, but not in HCoEpiC cells

ATR generally responds to replicative stress while ATM and DNA-PK respond to the repair of dsDNA breaks. However, there are studies showing the overlap between these pathways. One of the markers of dsDNA breaks are the increased phosphorylation of γH2AX(S139P) [69, 73, 74], which is also regulated by ATR [74]. Inhibition of Chk1 by gemcitabine and 5-FU treatments has been shown to increase γH2AX(S139P) levels through ATR-mediated replicative stress [74, 75].

**Figure 8:**
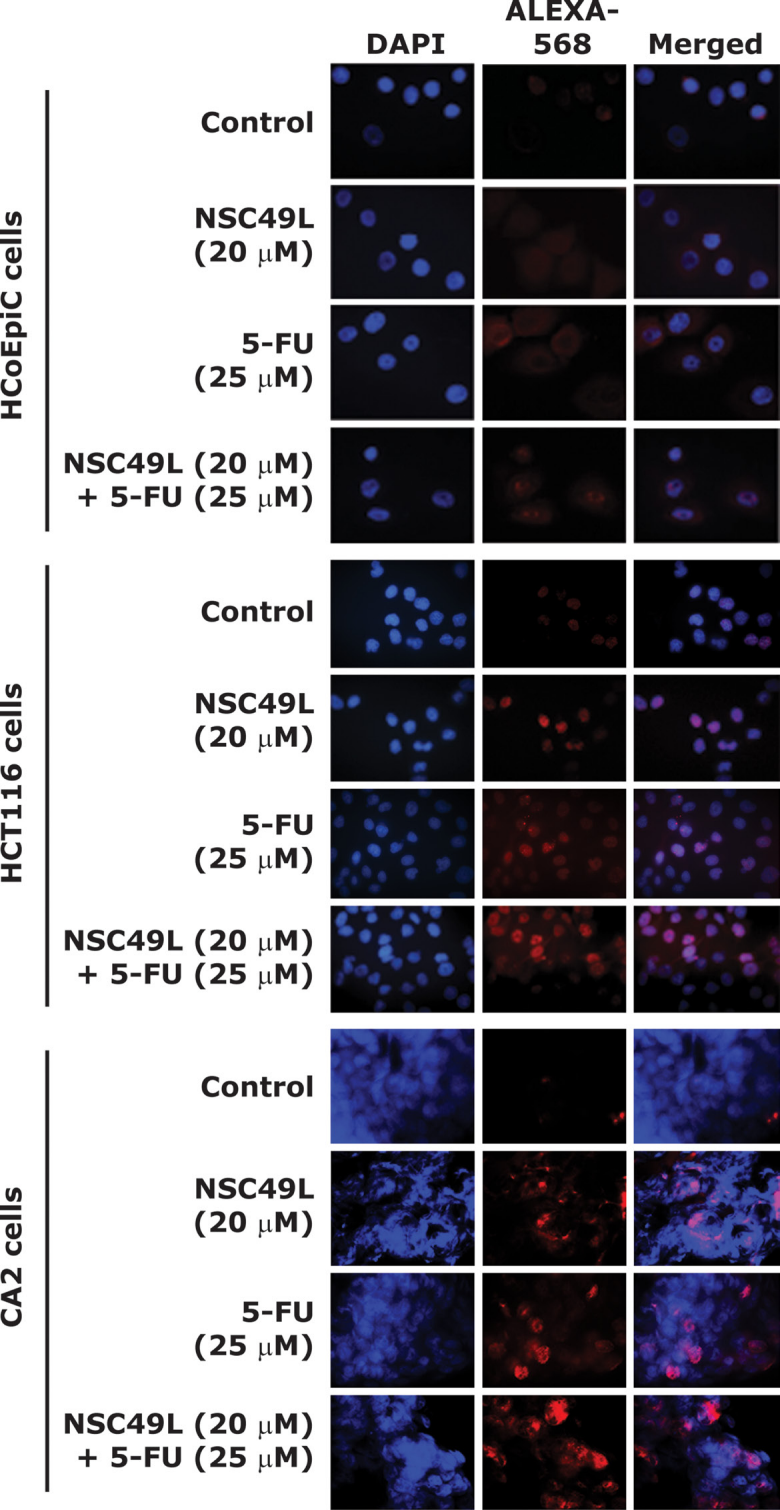
NSC49L and 5-FU treatments increase γH2AX(S139P)-foci IHC staining showing γH2AX(S139P)-foci formation (red) in nuclei of HCoEpiC, HCT-116 and CA2 cell lines treated with different concentrations of NSC49L and 5-FU either alone or in combination for 24 h. Data are representative of three different experiments. Magnification = 40×.

In the present study, we determined whether NSC49L- and 5-FU-induced cytotoxicity was related to DNA-strand breaks and phosphorylation of H2AX, by examining the levels of γH2AX(S139P) by immune-histochemical (IHC) staining. Our results showed increased γH2AX(S139P)-foci after treatment with NSC49L alone in HCT-116 and CA2 cells. The γH2AX(S139P)-foci were further increased in combination with 5-FU in both HCT-116 and CA2 cells, but not in HCoEpiC cells (Figure 8). Previous Chk1 inhibitors UNC-1, Cep-3891, MK8776, MK-1775, AZD7762, LY2603618 and SCH900776 also induce γH2AX(S139P)-foci as single or combined agent treatments in cancer cells [69, 76–80]. Together, our results suggest that NSC49L either alone or in combination with 5-FU induces replication stress-mediated DNA damage in HCT-116 and CA2 cells, but has no effect on normal colonic epithelial cells, HCoEpiC, which is a desirable feature for therapeutic development.

The activity of the ATR/Chk1 pathway is important for stabilizing stalled forks as well as to activate S-G_2_/M-phase checkpoints to prevent the entry of unreplicated DNA into mitosis [81]. However, once the ATR/Chk1 pathway is inactive and the dNTP pools are exhausted, replicative stress increases with an increase of non-progressive replication forks [82]. Ultimately, the amount of ssDNA exceeds the amount of available RPA and the collapsed replication forks lead to generation of dsDNA breaks [33, 82], mitotic catastrophe, and cell death [38, 47, 82, 83].

### Pharmacokinetics of NSC49L

In moving our studies into an animal model, we first determined the pharmacokinetics of NSC49L in male CD1 mice as described in Table 2. The t_1/2_ of NSC49L was 0.234 ± 0.098 h, which is similar to 5-FU (0.21 ± 0.06 h) [84]. However, the bioavailability of NSC49L (AUC = 1.306 ± 0.086 h*μg/ml) (Table 2) is lower than 5-FU (59.71 ± 10.58 h*μg/ml) [84]. This implies that a daily dosing regimen of NSC49L may be required to maintain the clinical concentration in the plasma. These experiments guided the PDTX experiments, as discussed below.

**Table 2:** Pharmacokinetic profile of NSC49L in CD1 mice

t_1/2_ (h)	t_max_(h)	C_max_(ng/ml)	AUC_last_(h*ng/ml)	AUC_Inf_(h*ng/ml)	AUC_%Extrap_obs (%)	AUC_last_/D (h*mg/ml)
0.234 ± 0.098	0.083	5530	1306 ± 86	1309 ± 89	0.187 ± 293	65.3 ± 4.3

NSC49L was dissolved in PBS and injected in male CD1 mice at 20 mg/kg body weight. Blood samples were collected at 0, 0.083, 0.25, 0.5, 1, 2, 4, 8 and 24 h. The pharmacokinetic profile of NSC49L was made by using LC/MS/MS (API-4000). Data are mean ± SD of three animals. t_1/2_ = elimination half-life, t_max_ = peak time, C_max_ = peak concentration, AUC = area under the plasma concentration-time curve, AUC_last_ = AUC from the time zero to the time of the last measurable concentration, AUC_Inf_ = AUC from time zero extrapolated to the infinite time, AUC_%Extra p = percentage of AUC_inf_that is due to extrapolation from AUC_last_ to the infinite time, AUC_last_/D = AUC_inf_ divided by the administered dose.

### Maximum tolerated dose (MTD) of NSC49L

**Figure 9:**
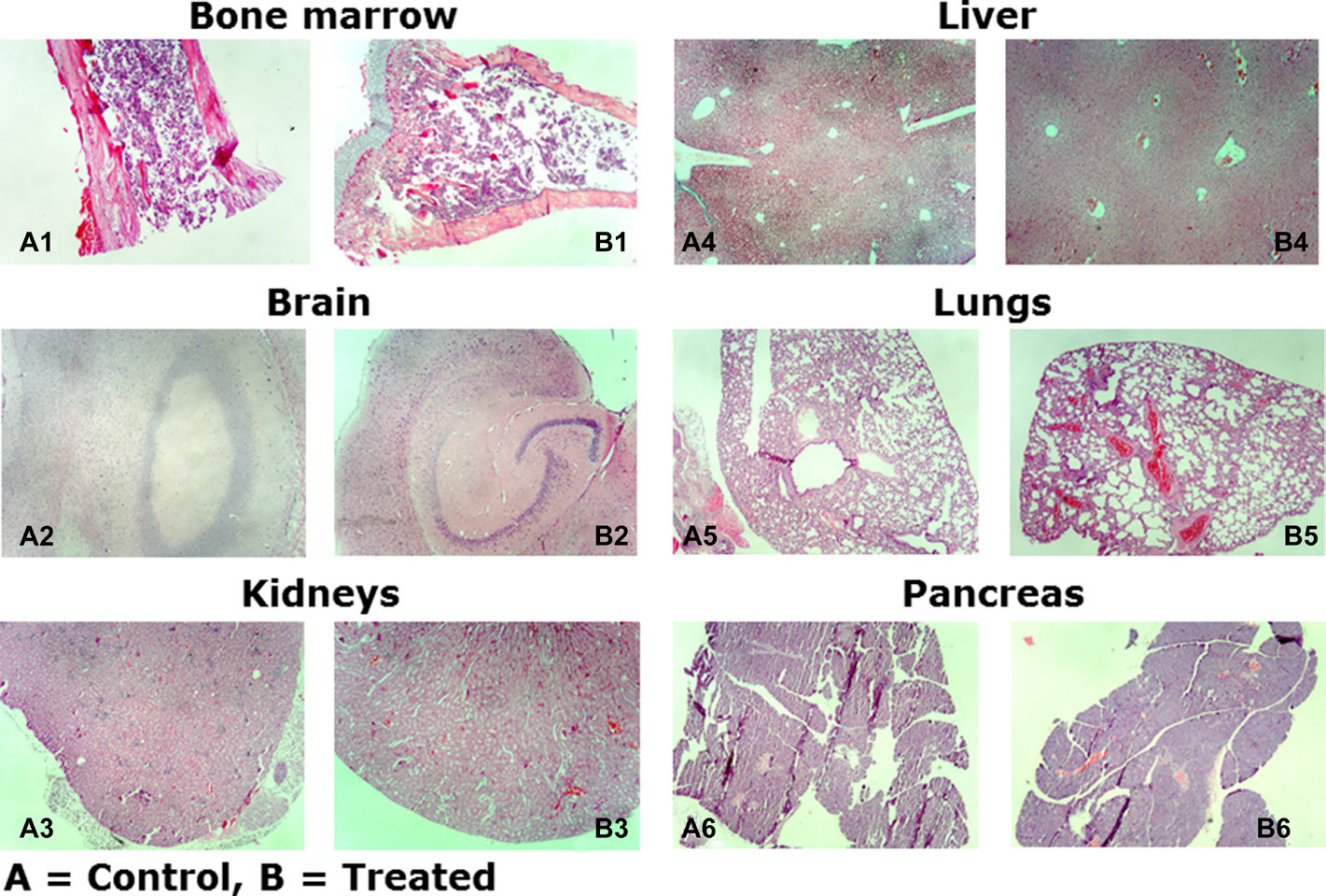
Histologic examination of NSC49L on SCID mice organs Tissue samples were formalin-fixed, paraffin-embedded, and examined with hematoxylin and eosin. Magnification: 100×.

In these experiments, mice were treated with 25, 50, and 75 mg/kg body weight for 5 consecutive days and followed by observation for an additional 7 days. There was no change in body weight, behavior, or pathological abnormalities in the treated versus control animals. These results suggest that this drug has a very good safety profile up to 75 mg/kg body weight. Assessment of the histologic sections revealed no significant differences between control and treated mice (Figure 9). Examination of the bone marrow reveals normal cellularity without fibrosis or decreased hematopoiesis between groups (Figure 9A1 and 9B1). The brain reveals no evidence of hypoxic-ischemic changes or significant neuronal loss in the cerebellum, hippocampus, and cortex, and the white matter appears unremarkable without significant gliosis (Figure 9A2 and 9B2). The kidneys demonstrate preserved tubules without evidence of acute tubular necrosis or glomerular injury (Figure 9A3 and 9B3). The liver demonstrates preserved portal and central areas without areas of central necrosis, inflammation, or significant fibrosis (Figure 9A4 and 9B4). There is mild vascular congestion observed in the treated kidneys and liver compared with the control group; however, this may be secondary to surgical handling of the tissues. The lungs appear aerated without evidence of acute lung injury, significant inflammatory infiltrates, or infarction (Figure 9A5 and 9B5). The pancreas appears histologically unremarkable (Figure 9A6 and 9B6). These results demonstrate a favorable safety profile of NSC49L in an *in vivo* mice model.

### Efficacy of NSC49L and 5-FU on human-derived CRC stem cell tumors *in vivo*

The high failure rate of new therapeutic agents in oncology is well recognized with the lack of pre-clinical models that recapitulate the heterogeneity of tumors in patients often cited as one plausible explanation [85]. To address this concern, the use of patient-derived tumor xenografts (PDTX) engrafted into immune-compromised mice has been suggested for preclinical modeling. This model has been widely accepted for CRC studies [86]. Since the PDTX model involves transplantation of cancer patient tissues/cells directly into immune-compromised mice, the genetic information and histological markers closely mimic the patient. Thus, the information obtained from the PDTX studies can be applied for the evaluation of new therapeutic drugs [87]. Furthermore, the outcome of the PDTX studies can be applicable for personalized cancer therapies.

**Figure 10:**
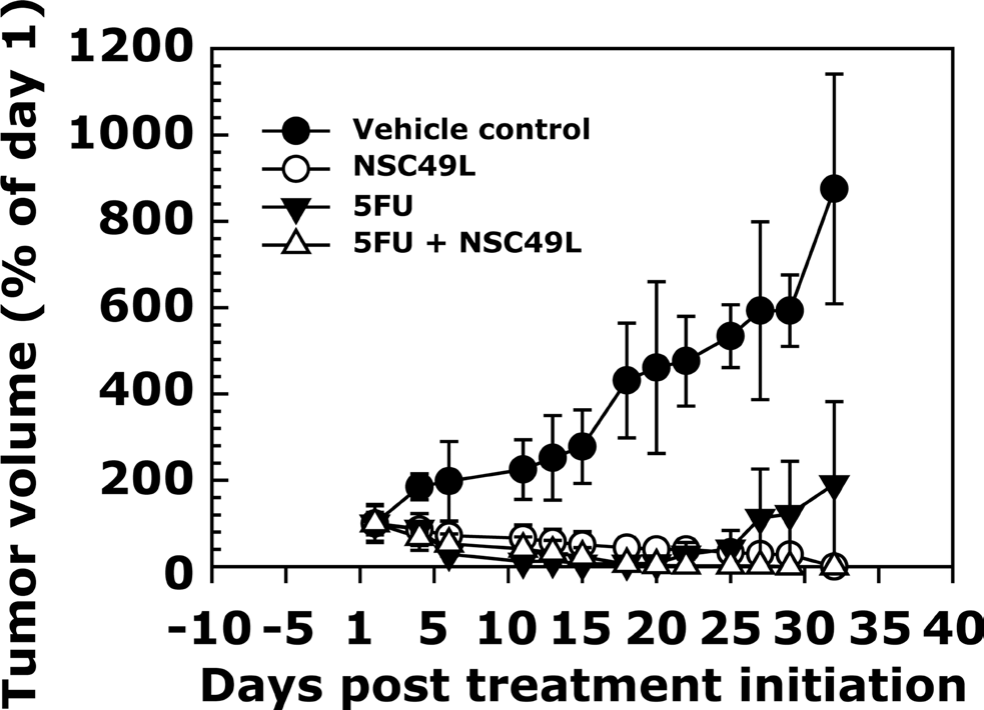
*In vivo* sensitivity of CRC stem cell tumors to NSC49L alone or in combination with 5-FU in a PDTX model The patient-derived CRC stem cell tumors were grown in female SCID mice and treated for 30 days either alone or in combination with NSC49L and 5-FU. The tumor volumes are shown in this graph as a percent of day 1. Data are mean ± SE of four animals in each group.

Currently the relationship between cancer stem cells, chemo-resistance, and metastasis is well-recognized in CRC patients [88, 89]. Therefore, applying PDTX model with CRC stem cell-like tumors may provide better information about the therapeutic usefulness of drugs to CRC patients. Thus, in the present study we determined the efficacy of NSC49L and 5-FU treatments either alone or in combination for the growth inhibition of human CRC tumors with stem cell like properties *in vivo* by using a PDTX model. Since the t_1/2_ of the NSC49L is short (0.234 ± 0.098 h, Table 2), we treated mice every day to maintain the pharmacological level of the drug in plasma. Based upon the MTD data, each day we treated mice intraperitoneally with 50 and 20 mg/kg body weight of NSC49L and 5-FU, respectively, for 30 days. The 5-FU treatment was also done every day but at a 2-fold lower dose than used in other animal studies [90]. The results showed a significant growth inhibition of CRC stem cell tumors with NSC49L and NSC49L combined with 5-FU treatment groups (Figure 10). Interestingly, the effect of 5-FU treatment alone was initially appreciable; however after the 21st day of treatment growth of the tumors resumed, but did not in those animals treated with the combination therapy. These results indicate the development of resistance to 5-FU (but not with the combination) by the CRC stem cells, which are consistent with previous findings and clinical observations [91].

Taken together, these results support the rational development of the orphan compound, NSC49L, as an innovative therapeutic agent affecting the Chk1 pathway with favorable traits including limited tissue toxicity, effectiveness in combination with 5-FU, and treatment resistance reversal. By impacting the self-renewal capacity of CRC stem cells, mitigating inherent treatment resistance, and inhibiting the growth of PDTX stem cell tumors, the incidence of tumor metastases and recurrence can be reduced or delayed and survival can be improved independent of genetic sub-types. These results justify further preclinical validation and clinical development.

## MATERIALS AND METHODS

### Ethics statement

All procedures performed in this study have been in accordance with the ethical standards of the institutional and/or national research committee and with the 1964 Helsinki declaration and its later amendments or comparable ethical standards.

### Cell lines and cell cultures

Human colon cancer cell lines HCT-116, LoVo, SW480, LS174T, RKO and HT-29 were obtained from the American Type Culture Collection (ATCC, Rockville, MD, USA). Cells were maintained as recommended by ATCC either in McCoy’s 5a or Dulbecco’s modified Eagle medium (DMEM; 4.5 g/L D-glucose) supplemented with 10% FBS and 1% antibiotic/antimycotic in tissue culture flasks in a humidified incubator at 37°C in an atmosphere of 95% air and 5% carbon dioxide. The medium was changed two times a week and cells were passaged using 0.05% trypsin/EDTA. Normal human colonic epithelial cell line, HCoEpic, was purchased from ScienCell Research Laboratories, Carlsbad, CA. These cells were grown according to manufacturer’s instruction. The isogenic HCT116(P53^+/+^) and HCT116(p53^−/−^) cell lines were a generous gift from Dr. Bert Vogelstein (Johns Hopkins, Baltimore, MD, USA).

In some experiments, 5-FU, leucovorin and oxaliplatin (FOLFOX)-resistant-HCT-116 and FOLFOX-resistant-HT29 cells were used. These cells were obtained from Dr. Adhip P. Majumdar (John D. Dingell VA Medical Center, Detroit, MI) [19]. These cells were generated by exposure to clinically relevant doses and schedules. The exposing schedule was for 12 cycles; each cycle lasted for one week. Briefly, the cells were first exposed to FOLFOX (25 μM 5-FU and 0.625 μM oxaliplatin) for 72 hours. The surviving cells were then cultured in normal medium without the drugs for 4 to 5 days. This cycle was repeated 12 times. The surviving cells were then split and exposed to higher doses of FOLFOX (50 μM 5-FU + 1.25 μM oxaliplatin or 100 μM 5-FU + 2.5 μM oxaliplatin) for 2 to 3 days per week for approximately 4 weeks. Finally, the resistant cells were maintained in normal culture medium containing a low dose of FOLFOX (5 μM 5-FU + 0.125 μM oxaliplatin).

CRC stem cell line, CA2, was obtained from Dr. Emina Huang (Lerner Research Institute, Cleveland, OH, USA). These cells were characterized for the expression of ESA^high^, ALDH1^+^, CD44^+^and CD133^+^ stem cell markers [20, 21]. These cells were maintained in DMEM/F12 (50/50 1×) medium containing, 6 g/ml D-glucose, 1 mg/ml NaHCO_3_, 4 mg/ml bovine serum albumin, 2 mM glutamate, 25 mg/ml ITS (insulin, transferrin, and selenium), 20 nM progesterone, 9.6 μg/ml putrescine, and 1% antibiotic/antimycotic solution in ultra-low attachment 6-well plates. Cultures were maintained in a humidified incubator at 37°C in an atmosphere of 95% air and 5% carbon dioxide.

### 5-fluorouracil (5-FU)

5-FU (99% HPLC purified) was purchased from Sigma-Aldrich (Cat No. F6627; St. Louis, MO, USA).

### NSC30049 (NSC49L)

NSC49L was synthesized in our laboratory following the procedure of Warmus *et al*. [16]. The final product was purified, crystallized, and characterized on the basis of NMR and mass spectral data. The purity was determined to be at the level of ≥ 99%.

### Cell survival assay

The survival of cells was determined by MTT (3-(4,5-Dimethylthiazol-2yl-)-2,5-diphenyl tetrazolium bromide) assay (ATCC, Manassas, VA, USA). In principle, the viable cell number is directly proportional to the purple formazan color of the reduced MTT dye, which can be quantitatively measured by spectrophotometry. Briefly, 1,500 cells were plated in quadruplets in 96-well flat-bottom tissue culture plates. After treatment with compounds for certain periods as described in respective figure legends, 10 μl of MTT reagent was added to each well and incubated at 37°C for 4 h to allow the formation of purple color crystals of formazan. In total, 100 μl of detergent solution was added to each well, and the reaction mixture was incubated in dark for 2–4 h at room temperature. The developed color density was then measured spectrophotometrically at 570 nm using the POLARstar Omega micro-plate reader (BMG Labtech, Inc., Cary, NC, USA).

### Spheroid formation assay

Live CA2 cells (100) were seeded in 384-well ultra-low attachment plates. Next, day cells were treated with different concentrations of NSC49L and 5-FU either alone or in combination. Cells were incubated for 7 days and the number of spheroids were counted in control and treated groups under an Olympus inverted microscope with a 10× magnification.

### Fluorescence-activated cell sorting (FACS) analysis

For determining the cell cycle profile, cells were plated in 60 mm tissue culture dishes and grown until 60% confluence. Cells were treated with 25 μM 5-FU for 24 h followed by treatment with 20 μM NSC49L for an additional 8 h. After treatment, cells were harvested at different time intervals, washed with ice-cold PBS and processed for FACS analysis as described previously [92, 93]. The ranges for G_0_/G_1_, S, and G_2_/M phase arrested and sub-G_1_ apoptotic cells were established on the basis of the corresponding DNA content of the histograms. At minimum, 10,000 cells were counted from each sample.

### CRC stem cell gene marker expression profiling by quantitative PCR

Total RNA was isolated from control and treated cells with RNAzol reagent (Invitogen, Carlsbad, CA, USA). The expression of mRNA levels of stemness genes was determined by qRT-PCR using Bio-Rad CFX96 Real Time System C1000 Thermal Cycler. The primers used for amplification of mRNA levels of genes were from Celprogen, Inc., as shown here with their Gene Bank accession and catalog numbers: *ALDHA1* (NM_000689, Cat. No. CepALDHA1-02), *Klf4* (NM_004235, Cat No. CepKlf4-02), *Nanog* (NM_024865, Cat. No. CepNanog-02), *Oct4* (NM_002701, Cat No. CepOct4-02), *Sox2* (NM_003106, Cat. No. CepSox2-02), *c-Myc* (NM_002467, Cat. No. Cepc-Myc-02) and *GAPDH* (NM_002046, Cat. No. Cep-GAPDH-02).

### Western blot analysis

The levels of various proteins were determined by Western blot analysis with our previously described procedure [94, 95]. The antibodies for phospho-Chk1(Ser317), phospho-Chk1(Ser317), phospho-Chk1(Ser319), phospho-Chk1(Ser345), Chk1, and GAPDH were purchased from Cell Signaling Technology (Danvers, MA, USA).

### Pharmacokinetic analysis

Pharmacokinetic analysis of NSC30049 in CD1 mice was determined by Pharmaron, Inc. (Louisville, KY, USA). Mice were injected intraperitoneally (IP) with NSC49L (20 mg/kg body weight) and approximately 0.04 ml blood was collected at 0, 5, 15 and 30 min, 1, 2, 4, 8 and 24 h time point. Blood of each sample was transferred into plastic micro-centrifuge tubes containing 2 μl of Heparin (1,000 IU). Samples were stored in a freezer at −75 ± 15°C prior to analysis. Concentrations of NSC49L in the plasma samples was analyzed using a LC-MS/MS method. WinNonlin^®^ (Phoenix^®^, version 6.1) software was used for pharmacokinetic calculations. The following pharmacokinetic parameters were calculated from the plasma concentration versus time data: T_1/2_, C_max_, AUC_last_, AUC_inf_, AUCextra%, Number of Points for Regression. The pharmacokinetic data is described using mean ± standard deviation (SD) and *n* = 3.

### Determination of maximum tolerable dose (MTD) and pathological analysis of organs

We have determined the MTD of NSC49L from male homozygous CB17 *scid/scid* (SCID) mice obtained from Tacomic Farms (Germantown, NY, USA). The mice were treated subcutaneously at 25, 50, and 75 mg/kg body weight for 5 consecutive days followed by observation for an additional 7 days. Every day body weight was recorded and behavioral changes were monitored in the treated versus control animals. After the 7th day animals were sacrificed and the organs from the mice treated at 75 mg/kg body weight were processed for pathological examination. Different organs were fixed in 10% buffered formalin, then sectioned and processed into paraffin blocks. Tissue sections were cut 4–5 μm thick and stained with hematoxylin and eosin. Histologic differences between control (A) and treated (B) mice were recorded and photomicrographs were taken with Olympus, BX41 microscope with attached camera.

### Patient-derived xenograft studies

With the pharmacokinetics analysis and MTD data in hand, we determined the efficacy of NSC49L and 5-FU alone and in combination for the growth of human-derived CRC stem cell tumors *in vivo*. CRC stem cells were isolated and characterized from the freshly resected human CRC tumor. One thousands of these CRC stem cells were injected subcutaneously into the right flank of the four 5-week old female SCID mice. After 20 days the first generation tumors (F_0_) of approximately 2,000 mm^3^ in size were harvested. They were divided into four equal pieces and implanted under the skin of the female SCID mice for the growth of second generation tumors (F_1_) for testing the drug efficacy studies. We divided the F_1_ tumor bearing mice into four groups and treated them every day subcutaneously with drugs as follows: i) PBS control, (ii) 50 mg/kg body weight NSC49L, (iii) 20 mg/kg body weight 5-FU, and iv) 50 and 20 mg/kg body weight NSC49L and 5-FU, respectively, for 30 days. The treatment with drugs began after 10 days of tumor growth. The body weight and tumor volumes were recorded twice a weak. The experiment was terminated on the 32nd day.

### Statistical analysis

All experiments were repeated at least three times and results were expressed as mean ± SE. One-way analysis of variance (ANOVA) was calculated with Sigma-Plot 9. A one-tailed *t*-test was used to compare any significant differences between control and treated groups. The criterion for statistical significance was *p* < 0.05. For western blotting data, band intensities were measured using ImageJ and normalized with GAPDH.
